# Concurrent adaptation to four different visual rotations

**DOI:** 10.1007/s00221-012-3150-4

**Published:** 2012-07-10

**Authors:** Monika Thomas, Otmar Bock

**Affiliations:** Institute of Physiology and Anatomy, German Sport University Cologne, Am Sportpark Müngersdorf 6, 50933 Cologne, Germany

**Keywords:** Motor learning, Sensorimotor adaptation, Contextual cueing, Visual rotation

## Abstract

The human sensorimotor system can concurrently adapt to two different distortions without interference when the distortions are cued by different contexts. We investigated whether this holds with four distortions as well. Subjects were exposed to an interlaced sequence of +30°, −30°, +60°, and −60° visuomotor rotations as the adaptation phase, cued by combinations of workspace location and by the arm used. Adaptation phase was followed by two episodes in each condition without any distortion testing the aftereffects. Results showed that the error at the onset of adaptation gradually decreased during adaptation to all four distortions without any sign of interference between the conditions. Furthermore, aftereffects of adaptation to ±30° rotation were significantly greater than of adaptation to ±60° rotation. We conclude that the human sensorimotor system is able to concurrently adapt to four different visual distortions when they are cued by different contexts. However, the results of aftereffects are ambiguous: Recalibration could be based on at least four parallel modules.

## Introduction

It is well established that our sensorimotor system adapts to a range of distortions in the environment, such as rotated visual feedback (Bock et al. 2003; Krakauer et al. 1999; Tong et al. 2002) and robotic force fields (Shadmehr and Mussa-Ivaldi 1994; Tong et al. 2002). When the sensorimotor system is exposed first to distortion A and then to a distinct distortion B, it is found that the two adaptive processes are not independent: Adaptation to A facilitates the subsequent adaptation to B when both distortions act in the same direction (Abeele and Bock 2001; Lazar and Van Laer 1968; Thomas and Bock 2010; Wigmore et al. 2002), but it interferes with the adaptation to B when both act in opposite directions (Krakauer et al. 2005; Thomas and Bock 2010; Wigmore et al. 2002). This led us to conclude that adaptation to different distortions can be based on one common process (Bock et al. 2003) or on several cooperative processes (Thomas and Bock 2010). Additional evidence for this view is provided by experiments that documented the transfer of adaptation to different movement types (Abeele and Bock 2003; Bock 2005), limbs (Freedman 1968; Hamilton 1964; Imamizu and Shimojo 1995; Sainburg and Wang 2002) as well as eyes and arm (Bock et al. 2008; Cotti et al. 2007).

The observed communality of adaptation seems to be facultative rather than obligatory, since subjects can concurrently adapt to two opposite distortions without any sign of interference if one prerequisite is met: The two distortions must be coded by adequate contextual information such as the arm used (Bock et al. 2005; Prablanc et al. 1975), movement direction (Pearson et al. 2010), initial arm posture (Gandolfo et al. 1996; Ghahramani and Wolpert 1997), target location (Woolley et al. 2007), or serial order (Welch et al. 1993). The suitability of audiovisual cues is controversial in literature (Osu et al. 2004, see however Gupta and Ashe 2007; Hinder et al. 2008; Woolley et al. 2007). Dual adaptation has been observed not only during exposure to the distortions, but also during the subsequent aftereffect phase, that is, the two opposite-adapted arms (Bock et al. 2005) or workspaces (Woolley et al. 2007) also produced opposite aftereffects. This is an important finding, since the processes at work during and after exposure to a distortion are probably not the same: Changes of performance during exposure are thought to reflect not only a recalibration of sensory-to-motor transformation rules but also strategic adjustments such as postural changes and anticipations, while aftereffects are thought to reflect recalibration alone (Bock 2005; Clower and Boussaoud 2000; McNay and Willingham 1998; Redding and Wallace 1996). If so, the existence of dual aftereffects would imply that concurrent adaptation is not merely a strategic phenomenon, but rather involves recalibration as well. More specifically, it has been suggested that adaptive recalibration is achieved by a common fast process and by multiple parallel slow processes that can be engaged in dependence on contextual cues (Lee and Schweighofer 2009).

Summing up the present state of our knowledge, it appears that multiple visual distortions can activate either common or distinct processes for adaptive recalibration, depending on the presence or absence of adequate contextual cues. Common processes manifest as transfer, facilitation or interference, and distinct processes as multiple adaptation. It should be noted, however, that experimental evidence for *multiple* adaptation is actually limited to *dual* adaptation: Previous work has shown that subjects can successfully adapt to two distortions, thus supporting the existence of two parallel processes, but not whether they can adapt to more than two distortions, which would support the existence of more than two parallel processes. The aim of this study was to test whether more than two processes can coexist in our sensorimotor system when they were cued by different context. To investigate, we let subjects concurrently adapt to four visual rotations that differed concerning their rotation direction and their rotation magnitude and were coded by a combination of two contextual cues, arm used, and target location. Aftereffects of all four conditions were tested by a de-adaptation phase at the end of the experiment. Hence, we can differentiate if recalibration of all four conditions occurs or if only different strategies were used.

## Methods

16 right-handed subjects (8 male, 8 female, 21–29 years of age) participated in this study.^1^ All reported to be free of sensorimotor dysfunctions except corrected vision, and none had prior experience with adaptation research. Those who presented with eye glasses continued to wear them throughout the experiment. All subjects signed an informed consent statement before participating in this study, which was part of an experimental program pre-approved by the authors’ institutional Ethics Committee.

**Fig. 1:**
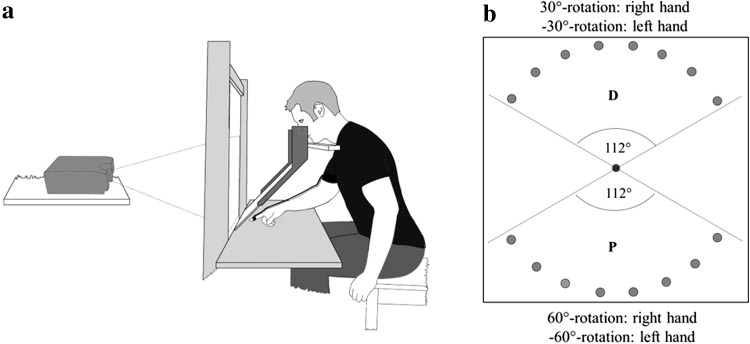
**a** Scheme of the experimental setup, with the mirror and the working surface. **b** Subjects’ view with computer-generated targets in 16 possible directions (only one target visible at a given time). Eight targets were presented at the distal half (*D*) and 8 targets on the proximal half (*P*) of the working surface

As shown in Fig. 1a, subjects sat and viewed a computer screen through a mirror, such that its virtual image appeared on a horizontal working surface. Subjects pointed with their right or left index finger from a central starting dot to one of eight sequentially presented targets and back to the center. Center and targets had a diameter of 1 cm and stayed on for 700 ms (targets) or until reached by the finger (center). Targets were located 10 cm from the center 16° apart and thus covered a range of 112°, either on the proximal or on the distal half of the working surface (D and P in Fig. 1b, respectively). Direct vision of the hand was prevented by the mirror. However, the position of the pointing index fingertip was registered by the Fastrak^®^ motion analysis system with a resolution of 1 mm and a sampling rate of 60 Hz and was displayed as a cursor on the computer screen along with the targets, thus providing visual feedback. The subjects were instructed to produce fast, straight, and uncorrected movements over the working surface, out to the target and back to the starting position.

The experiment was subdivided into 84 episodes of 30-s duration, separated by rest breaks of 5 s. Each episode allowed the execution of about 20 pointing movements^2^ under one of the following conditions.

- DR0: distal targets, right hand, unrotated feedback
- DL0: distal targets, left hand, unrotated feedback
- PR0: proximal targets, right hand, unrotated feedback
- PL0: proximal targets, left hand, unrotated feedback
- DL−30: distal targets, left hand, feedback rotated by −30° about the center
- PL−60: proximal targets, left hand, feedback rotated by −60° about the center
- DR+30 distal targets, right hand, feedback rotated by +30° about the center
- PR+60: proximal targets, right hand, feedback rotated by +60° about the center.

Thus, hand and target location served as cues about the distortion.

Subjects started with a baseline phase of eight episodes, two each in condition DR0, DL0, PR0, and PL0; the order of conditions was counterbalanced across subjects. Next came the adaptation phase of 68 episodes, where the conditions DL−30, PL−60, DR+30, and PR+60 were presented in an interlaced sequence: Each condition was administered once in each block of four episodes, in an order that was counterbalanced across subjects. The experiment concluded with a de-adaptation phase of eight episodes, two each in condition DR0, DL0, PR0, and PL0, again counterbalanced across subjects.

Data from the *adaptation phase* were analyzed by calculating the angular error of each movement 150 ms after its onset, an established parameter that minimizes the effects of feedback-based corrections (Bock and Thomas 2011; Werner et al. 2009). We also calculated the reaction time of each movement and determined the means of angular error and reaction time for each episode and subject. Mean angular error was then normalized as follows:

1 $$ n_{i,n} = \frac{{e_{i,k} }}{{R_{i} }} $$

*e*_*i,k*_ equals the above-baseline error in episode *i* of subject *k*, and *R*_*i*_ equals the feedback rotation in episode *i*. Mean reaction time was normalized by subtracting the baseline. The normalized scores were submitted to analyses of variance (ANOVAs), using the within-factor block and the between-factors magnitude of rotation (30°, 60°) and direction of rotation (+, −). The outcome was Greenhouse-Geyser adjusted when necessary, and significant effects were scrutinized with LSD post hoc tests.

Data from the *de*-*adaptation phase* were analyzed by calculating the magnitude of the aftereffect as follows:

2 $$ {\text{AE}}[\% ] = \frac{{{\text{De}}_{1,k} }}{{R - \overline{Ae}_{15 - 17},k}} \times 100, $$

where De_1,*k*_ equals the angular error of the first de-adaptation episode of subject *k* for a given condition, *R* equals the corresponding visual rotation, and $\overline{{Ae_{15-17} }}$ equals the mean angular error of adaptation episodes 15–17 of subject *k*. The outcome was submitted to an analysis of variances (ANOVA) with the between-factors magnitude and direction.

## Results

**Fig. 2:**
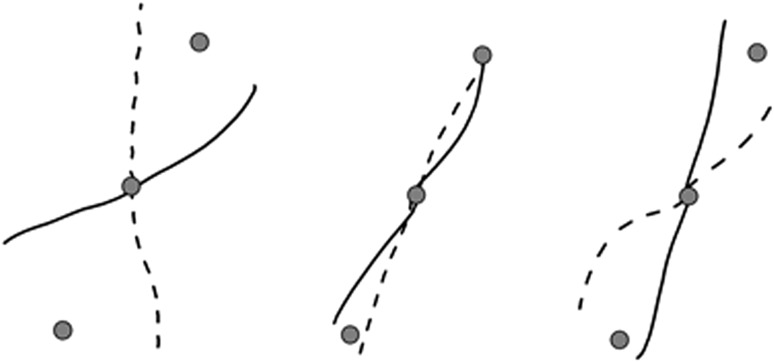
Cursor paths of one subject at the onset of adaptation, the end of adaptation and the onset of de-adaptation for all four conditions. *Dashed lines* represent the −30° rotation (distal half) and −60° rotation (proximal half) and the *solid lines* the 30° rotation (distal half) and 60° rotation (proximal half) condition

Figure 2 depicts original cursor paths of one subject during the first and last adaptation episode and the first de-adaptation episode for each of the four adaptation conditions. The large error at the onset of adaptation decreased distinctly by the end of adaptation, and a negative aftereffect emerged during de-adaptation for all four conditions.

**Fig. 3:**
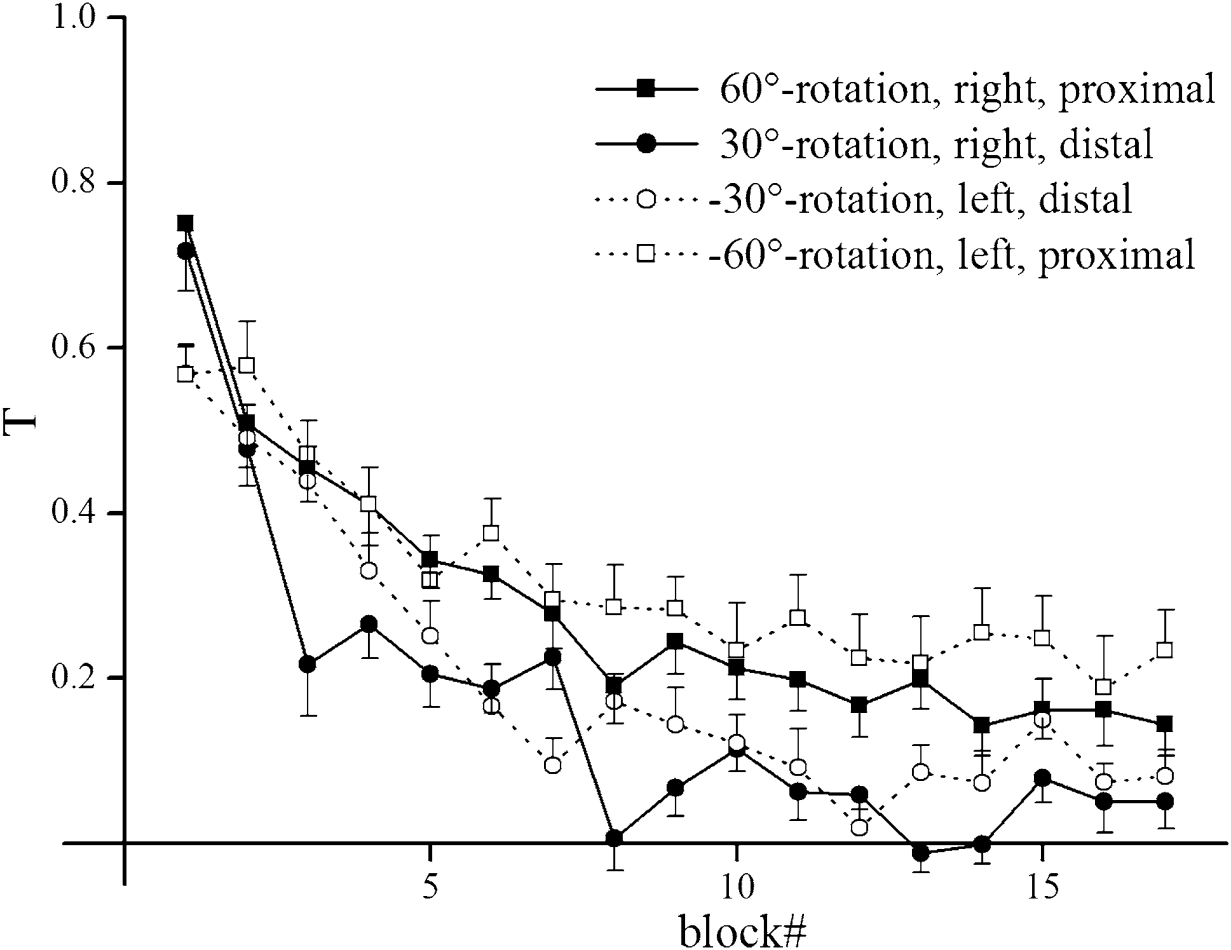
Time course of mean error during the adaptation phase separated for each adaptation condition. *Symbols* represent across subject means and *error bars* standard deviations. The legend*s* represent the rotation, the arm used, and the target location

Figure 3 illustrates the normalized angular error in each condition throughout the adaptation phase, averaged across subjects. Note that the abscissa enumerates blocks rather than episodes, and the four symbols represent the four conditions encountered in each block. Obviously, the initial error decreases gradually for all four adaptation conditions, but the decrease was smaller for adaptation to −60° and +60° rotation than for adaptation to −30° and 30° rotation. Accordingly, ANOVA yielded significant effects of block and magnitude and also for the interaction of both showing that the difference between magnitudes was more pronounced in later blocks of adaptation. Further, ANOVA yielded a significant effect for the interaction Block × Direction. The LSD post hoc test showed that errors differed between directions only in block 1 and 8. Additionally, we found a significant effect of the interaction Block × Magnitude × Direction. The LSD post hoc test showed that the difference between magnitudes was absent for some combination of block and direction and present for the first and third block between directions (Table 1).

**Table 1:** Statistical outcomes of the ANOVA of angular error during adaptation phase

Effect	*F* value	*p* value
Magnitude	*F*(1,64) = 23.32	*p* = 0.0000
Direction	*F*(1,64) = 0.04	*p* = 0.8328
Magnitude × Direction	*F*(1,64) = 0.01	*p* = 0.8512
Block	*F*(7.75, 496.23) = 156.59	*p* = 0.0000
Block × Magnitude	(*F*(7.75, 496.23) = 2.03	*p* = 0.0422
Block × Direction	(*F*(7.75, 496.23) = 5.94	*p* = 0.0000
Block × Magnitude × Direction	*F*(7.75, 496.23) = 2.52	*p* = 0.0116

**Fig. 4:**
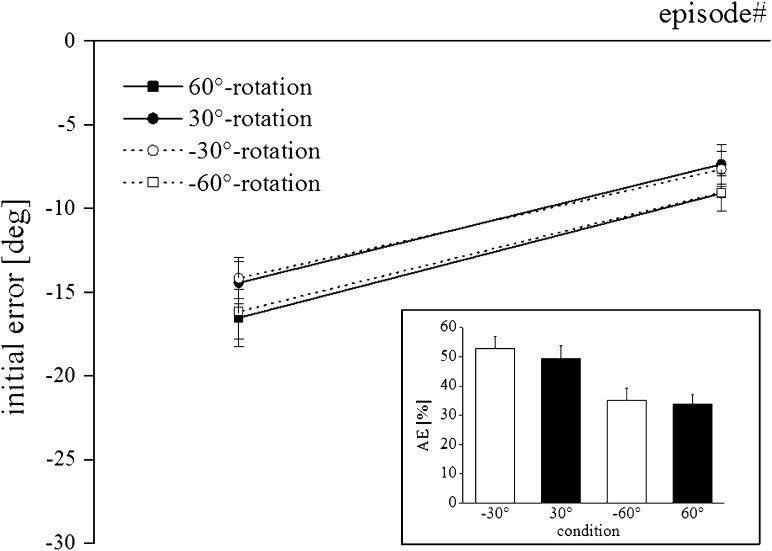
Time course of mean error during the de-adaptation phase separated for each de-adaptation condition. *Symbols* represent across subject means and *error bars* standard deviations. *Inset* The magnitude of the aftereffect for each adaptation condition

Aftereffects averaged 42 % across subjects and distortions. Figure 4 shows that aftereffects were about 50 % larger for 30° as compared to 60° rotations, irrespective of their direction. Accordingly, ANOVA yielded a significant effect only for magnitude (*F*(1,44) = 18.92; *p* < 0.001).

Data from both de-adaptation episodes are plotted without normalization in the main part of Fig. 4 and show that the absolute scores following 60° rotations are only marginally larger than those following 30° rotations. It becomes obvious that errors were substantially different from zero showing an opposite error compared to adaptation, indicating recalibration of adaptation for all conditions. ANOVA confirmed this observation by yielding a significant constant (*F*(1,44) = 424.24; *p* < 0.001). Further, ANOVA revealed no other significant effects. The results of the calculated aftereffects of each condition were plotted in the inset of Fig. 4 as percentage of adaptation magnitude. Clearly from this calculation, aftereffects from adaptation to a rotation magnitude of 30° were substantially greater than from adaptation to a rotation magnitude of 60°, while aftereffects from adaptation to different rotation directions were similar for the same rotation magnitude. Accordingly, ANOVA yielded a significant effect for the factor magnitude (*F*(1,44) = 18.92; *p* < 0.001) but not for the factor direction.

**Fig. 5:**
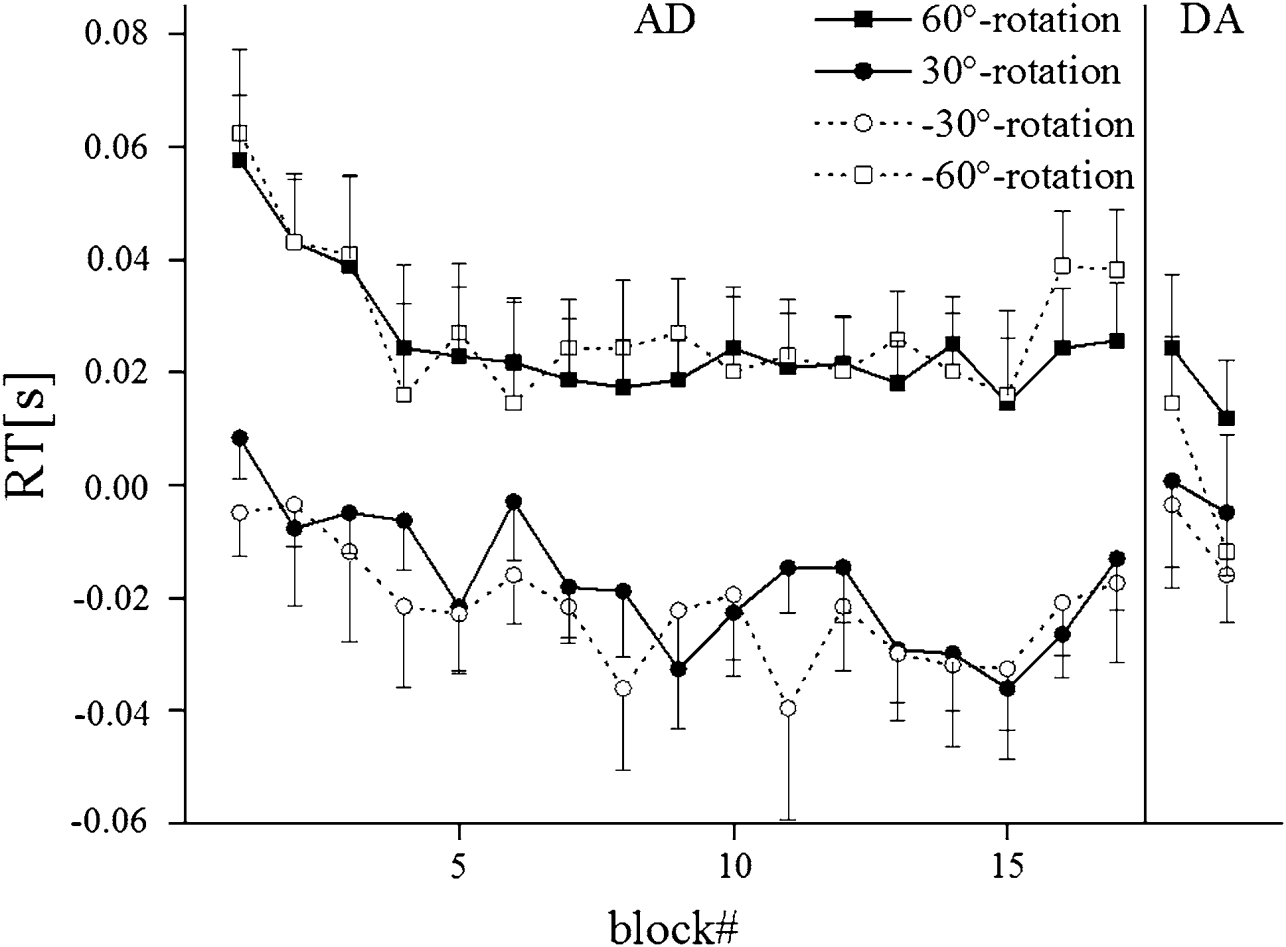
Time course of mean reaction time during adaptation (*AD*) and de-adaptation phase (*DA*) for each condition. *Symbols* represent across subject means and *error bars* standard deviations

Figure 5 demonstrates that the reaction time was higher when adapting to 60° as compared to 30° rotations, but this difference almost disappeared during de-adaptation. ANOVA of the adaptation phase (AD) yielded a significant effect of magnitude (*F*(1,44) = 28.8; *p* < 0.001), while ANOVA of the de-adaptation phase (DA) yielded no significant effects.

## Discussion

While several previous studies have dealt with concurrent adaptation to two distortions (see “Introduction”), the present work for the first time addresses the concurrent adaptation to four distortions, coded by the combination of two contextual cues: arm used and target hemispace. Our data confirm that either cue enables concurrent adaptation (Bock et al. 2005; Woolley et al. 2007). More importantly, it shows for the first time that concurrent adaptation is not limited to two distortions, but rather can encompass at least four different distortions.

After about eight episodes or 140 movements under each distortion, subjects’ performance reached a plateau that compensated for about 80 % of the distortion (i.e., normalized error was 0.2). Thus, the efficiency of adapting to four distortions was comparable to that observed with a single distortion in numerous earlier studies. Our data from the adaptation phase therefore provide no evidence for an interference between concurrent adaptive tasks, which fits well with the notion that adaptation is based on parallel modules (Ghahramani and Wolpert 1997; Lee and Schweighofer 2009).

In fact, different possibilities must be considered leading to this adaptation pattern. Besides the existence of four different adaptive representations, two distinct binary representations could explain this data as well, namely one representation varying between the two arms and the other between the two workspaces such that magnitude and directions are encoded separately. During adaptation, we found a significant difference between rotation magnitudes. Here, it has to be taken into consideration that the ±30° rotations were always presented in the distal and the ±60° rotations in the proximal half of the workspace such that the effect could also be due to the different hemispaces. In the study of Woolley et al. (2007), no difference in adaptation between different hemispaces became obvious. In contrast, when comparing single adaptation data to ±30° and ±60° rotation of our group, we found comparable differences during adaptation between both magnitudes such that it is obvious that differences are due to the rotation magnitude rather than the hemispace. Additionally, we found significant effects for interactions with direction (Block × Direction, Block × Magnitude × Direction). Due to the fact that only two episodes varied between the conditions without any regularity, these results seem to be chance results.

Due to the fact that aftereffects give information about the proportion of recalibration (see “Introduction”), the significant results of de-adaptation indicate successful recalibration for all four adaptation conditions. Further analyses show that in relation to error reduction during adaptation, the aftereffects were significantly greater for adaptation to the 30° rotations than to the 60° rotations. One could argue that the sensory-to-motor transformation rules of the adaptation to ±30° were recalibrated and accessed during all four de-adaptation conditions. In other words, error reduction during adaptation to the 60° rotation conditions could be based on the recalibration of the 30° rotation, and the offset between both distortions may be compensated by strategic components. To verify this consideration, we recalculated the magnitude of the aftereffect of the adaptation to ±60° by referring it to the adaptation to ±30° as follows:

$$ {\text{AE}}[\% ] = \frac{{{\text{De}}_{1,k} }}{{ \pm 30^\circ - \overline{{Ae_{15-17} ,k}} }} \times 100, $$

where De_1,*k*_ is the angular error of the first de-adaptation episode of subject *k* for ±60° condition, and $\overline{Ae}$ is the mean angular error of adaptation episodes 15–17 to ±30° rather than ±60° with the corresponding direction of subject *k*. The results show 56 % aftereffect for the 60° rotation and 60 % aftereffect for the −60° rotation, so 7–8 % greater effects than for the ±30° rotation. This finding argues against the above interpretation of only recalibration of the adaptation to the ±30° rotation. In fact, it seems that recalibration for all four adaptation conditions has occurred, less for the adaptation to ±60° than to ±30°, and both conspicuously smaller compared to recalibration of single adaptation. A single visual rotation produces aftereffects of about 70–80 % (Bock 2010; Mazzoni and Krakauer 2006; Miall et al. 2004), but the four concurrent rotations in the present study led to aftereffects of only about 40 %. It is interesting to compare this outcome to studies using two visual rotations: aftereffects averaged 62 % when distortions were cued by the arm used (Bock 2010) and about 45 % when they were cued by the hemispace (Woolley et al. 2007). When considered together, this pattern of findings suggests that the aftereffects of multiple adaptation show signs of interference, more so when distortions are cued by the hemispace rather than by the arm used.

Even though this interpretation is rather speculative, the mechanisms underlying independent adaptations can support it. Some authors assume that different muscle synergies must be involved to evoke independent adaptation such that different sensorimotor pathways are linked to the different adaptation conditions (Woolley et al. 2007). In our study, the different adaptation conditions are partially linked to different arms and therefore definitively linked to different sensorimotor pathways. Furthermore, we used different workspaces that could not completely be associated with different muscle synergies. Woolley et al. (2007) used different workspaces as contextual cues as well, but due to the different experimental design, different muscle synergies were involved. For contextual cues that are not associated with different muscle synergies, the results are inconsistent. While some studies fail to show dual adaptation with color cues (Hinder et al. 2008; Woolley et al. 2007), successful dual adaptation has well been shown with color cues (Osu et al. 2004; Wada et al. 2003) and different starting locations (Ghahramani and Wolpert 1997). However, adaptation took a considerably longer time in the study of Wada et al. (2003) than in other studies where distortions were linked to different muscle synergies (Bock et al. 2005, Gandolfo et al. 1996). In fact, studies show that functional cueing can also lead to independent adaptation but it seems that adaptation processes are more fragile or might be stronger based on strategies. With regard to the fact that in our experiment, the different rotation magnitudes were cued by the different workspaces; they were not distinguished by different muscle synergies. That might require more strategies and lead to interference between distinct adaptive states. As a further result, we found longer reaction times during adaptation for the rotation magnitude of 60° than of 30° rotation. In contrast, Anguera et al. (2007) found no difference in reaction times for adaptation to a 30° and a 45° rotation. The same holds for unpublished data from experiments of our research group, where no differences in reaction times for adaptation to a 30° and a 60° rotation became obvious. In conjunction with the difference in recalibration between the ±30° and ±60° condition, this could be taken as an indicator for greater strategic components during adaptation to ±60° rotation compared to ±30° rotation supporting the idea of interference with a greater influence on the more complex distortion.

Within this conceptual framework, our data suggest that quadruple adaptation produced less recalibration, but this decrement was evened out by a larger strategic component. Thus, summing up, recalibration could be based on multiple (at least four) parallel modules that can be easily linked to different arms, but not so easily to different hemispaces; during the adaptation phase, recalibration could be supplemented by strategies such as achieving a desired time course of adaptive compensation, which increases from 0 % to about 80 % of the distortion within about 140 movements.

Our interpretation, that adaptive modules cannot easily be linked to different hemispaces, implies that adaptation to visual rotations largely generalizes across the whole workspace. This seems to be at odds with the earlier observation that adaptation to eight targets distributed across the workspace is slower than that to a single target (Krakauer et al. 2000), a phenomenon related to the number of targets rather than their spacing (Bock and Schmitz 2011). However, the latter findings are based on data from the adaptation phase, while our present interpretation is derived from the aftereffects. The seemingly discrepant results can therefore be reconciled by postulating that the parallel modules for recalibration largely generalize across the workspace, while strategies are target specific. More research is desirable to better understand the interplay of recalibration and strategies throughout the adaptation phase.

Certainly, this study, which provided a basic experiment concerning multiple adaptation including concurrent adaptation to more than two different distortions does not claim to answer all questions relating to this context. In conclusion, we found that it is possible to concurrently adapt to more than two, namely four different visual distortions that are cued by different contexts. Further experiments are needed to address the consideration of the number of adaptive processes involved in multiple adaptation to more than two different distortions. Therefore, it would be necessary to change the mapping between the distortions and the contextual cues. In addition, changing the mapping is important to get further revealing explanations concerning the recalibration pattern resulting from this study.

## Footnotes

1 We tested for sex-related differences concerning all parameters which were used and found no significance.

2 The variability between episodes and conditions was 0.78 ± 0.64 movements and between subjects 0.82 ± 0.7 movements.
